# Polymorphism rs2066865 in the *Fibrinogen Gamma Chain* (*FGG*) Gene Increases Plasma Fibrinogen Concentration and Is Associated with an Increased Microvascular Thrombosis Rate

**DOI:** 10.3390/medicina55090563

**Published:** 2019-09-03

**Authors:** Karina Drizlionoka, Jānis Zariņš, Agnese Ozoliņa, Liene Ņikitina-Zaķe, Biruta Mamaja

**Affiliations:** 1 Department of Anaesthesiology, Riga East Clinical University Hospital, LV-1024 Riga, Latvia; 2 Centre of Plastic and Reconstructive Microsurgery of Latvia, LV-1024 Riga, Latvia; 3 Department of Anaesthesiology and Critical Care, Riga Stradins University, LV-1007 Riga, Latvia; 4 Latvian Biomedical Research and Study Center, LV-1067 Riga, Latvia

**Keywords:** fibrinogen, *fibrinogen gamma chain* gene, polymorphism, microvascular flap thrombosis, free flap failure, free tissue transfer

## Abstract

*Background and Objective*: Thrombosis due to inherited hypercoagulability is an issue that has been raised in microvascular flap surgery previously. We analyzed the association of a single nucleotide polymorphism (SNP) in rs2066865 in the *fibrinogen gamma chain* (*FGG*) gene, alteration in plasma fibrinogen concentration, and presence of microvascular flap thrombosis. *Materials and Methods*: A total of 104 adult patients with microvascular flap surgery were subjected to an analysis of the presence of SNP rs2066865 in the *FGG* gene. Alterations in plasma fibrinogen concentration according to genotype were determined as a primary outcome, and flap thrombosis was defined as a secondary outcome. *Results*: Flap thrombosis was detected in 11.5% of patients (*n* = 12). Successful revision of anastomosis was performed in four patients, resulting in a microvascular flap survival rate of 92.3%. We observed an increase in plasma fibrinogen concentration in genotype G/A and A/A carriers (G/G, 3.9 (IQR 4.76-3.04); G/A, 4.28 (IQR 5.38-3.18); A/A, 6.87 (IQR 8.25-5.49) (A/A vs. G/A, *p* = 0.003 and A/A vs. G/G, *p* = 0.001). Within group differences in microvascular flap thrombosis incidence rates were observed—G/G 6/79 (7.59%); G/A 5/22 (22.7%); A/A 1/3 (33.3%) (OR 0.30 95%; CI 0.044 to 0.57), *p* = 0.016; RR 3.2—when G/G versus G/A and A/A were analyzed respectively. *Conclusions*: A/A and G/A genotype carriers of a single nucleotide polymorphism in rs2066865 in the *fibrinogen gamma chain* gene had a higher plasma fibrinogen concentration, and this might be associated with an increased microvascular flap thrombosis incidence rate. Determined polymorphism could be considered as a genetic marker associated with microvascular flap thrombosis development. To confirm the results of this study, the data should be replicated in a greater sample size.

## 1. Introduction

Microvascular flap surgery poses an ability to cover a broad range of tissue defects for reconstructive purposes, fostering functional and aesthetic recovery. Flap thrombosis is still the leading cause of flap loss, resulting in patient and surgeon disaffection as well as increased hospital stay and costs. Overall, thrombotic events are attributable to 3% to 12% of flap complications. Although technical factors are of constant concern, inherited thrombophilia has been mentioned as a potential risk factor. Factor V Leiden, prothrombin gene mutation, protein C and protein S deficiencies, antithrombin deficiency, and mutation of the *methylene tetrahydrofolate reductase* gene causing hyper-homocysteinemia are mentioned in literature as contributing factors leading to flap thrombosis; therefore, preoperative thrombophilia screening could be a cost-effective tool for the prevention of microvascular flap thrombotic complications [1,2,3].

Thrombosis is a heterogeneous disorder [4]. Microcirculation in the flap is affected by a variety of factors. Hypercoagulation and thrombosis due to increased plasma fibrinogen concentration are still unclear. Both structural and functional defects of fibrinogen have been reported as risk factors for deep venous thrombosis [5], and few studies have described an increased risk of arterial thrombosis caused by fibrinogen abnormalities [6,7]. Fibrinogen also is viewed as an acute phase reactant. The fibrinogen level rises in relation to aging [8] and pathophysiological changes such as inflammation conditions [9], trauma, and malignancy [10,11,12].

Fibrinogen is a plasma glycoprotein with a molecular weight of 340 kDa and is synthesized by hepatocytes. Fibrinogen molecules are elongated structures 45 nm long, which are comprised of two sets of three polypeptide chains—A alpha; B beta; and gamma [13]. The three chains are encoded by three separate genes, *fibrinogen alpha* (*FGA*), *fibrinogen beta* (*FGB*), and *fibrinogen gamma* (*FGG*), clustered in a region of approximately 50 kb on chromosome 4q31.3 [14]. Polymorphism rs2066865 in the *FGG* gene is described as a reason for alterations in the coagulation system. Particularly, fibrinogen gamma H2 haplotype-tagging SNP (FGG 10034C > T) has been demonstrated as a risk factor for deep venous thrombosis [15]. Haplotype H2 is associated with reduced plasma fibrinogen ‘ levels, thus promoting resistance to fibrinolysis through unique binding sites for coagulation factor XIIIB. The γ′ chain is also reported to protect from inactivation by antithrombin, which is thought to facilitate heparin resistance to clot-bound thrombin and further contributes to thrombin activity on the clot surface, thus increasing the time for thrombus formation [16]. 

Therefore, we tested the hypothesis that SNP rs2066865 in the *FGG* gene alters the level of plasma fibrinogen concentration and could associate with an increase in the microvascular flap thrombosis rate.

## 2. Materials and Methods

### 2.1. Subjects

In this observational case series, we included a total of 104 patients who underwent microvascular flap transfer in The Centre of Plastic and Reconstructive Microsurgery of Latvia from 2016–2018.

We enrolled all adult patients undergoing microvascular flap surgery during the study period. The protocol and the informed consent form, including the request to donate genetic material, were approved by the Latvian Central Ethics Committee (Nr.1/28-11-16). All patients provided written, informed consent.

The exclusion criteria were: pregnancy, peripartum period; transfusion of allogeneic blood components, and/or coagulation factors within 72 h perioperatively; proven left ventricular failure; allogenic bone marrow transplantation; liver failure, liver transplantation; and end-stage kidney disease.

For patients on direct oral anticoagulants, medication was stopped 72 h prior to sample collection to avoid any inaccuracy in testing. For patients taking a vitamin K antagonist, medication was stopped, according to the patient’s international normalized ratio (INR) level, 4 to 5 d prior to sample collection. Patients with a high thrombosis risk were switched to low-molecular-weight heparin (LMWH) and discontinued use 12 h prior to the surgery. 

We determined SNP rs2066865 (G > A) in the *FGG* gene and collected laboratory data, including total plasma fibrinogen concentration, platelets, white blood count, and C-reactive protein.

An interview was performed to register patient histories, particularly with interest in any previous thrombotic event, use of antithrombotics, regular medication (including oral contraceptives), family history of thrombotic events, and any previously diagnosed inherited thrombophilias (factor V Leiden, prothrombin gene mutation, antithrombin deficiency, protein C deficiency, protein S deficiency, etc.) We registered all tissue injury causative factors and defined 30 d as a recent trauma period in case of trauma or polytrauma etiology.

In regards to demographics, history, and family history collection, patient interviews were performed by the same clinician. A positive history of thrombosis was defined as any thrombotic event with either arterial or venous origin. Group malignancy consisted of patients with orofacial tumors in different stages (i.e., active malignancy) after treatment with actinotherapy and palliation.

All patients received standardized general anesthesia. According to our local guidelines, administration of 10 mL/kg of Dextran 40 (Fresenius Kabi, Polska Sp. Warsaw, Poland) intravenously, at the time the anastomoses were assured, was performed. Patient temperatures were monitored during the surgery. We measured axillar and nasopharyngeal temperatures, and ∆*t* 1°C was considered as the optimal difference between core and peripheral temperature. Infusion of warm fluids and a warming blanket were used to prevent a drop in the core temperature. During the study, a total of 104 microvascular flap transfers were performed by highly trained specialists. The venue of thrombosis (e.g., arterial, venous, or both) was assured by direct visualization during the revision of anastomosis.

### 2.2. Laboratory Workup

Blood samples were drawn on the day of surgery prior to the induction of anesthesia and any crystalloid infusion. All tests were processed within an hour. 

Total plasma fibrinogen concentration was measured by the Clauss method [17] (normal range 2–4 g/L) in citrated plasma using the STA-R COMPACT (Diagnostika Stago, Asnières-sur-Seine, France). Platelet count (normal range 150–400 × 10^9^/L) was measured using the Sysmex XP-300 (Sysmex Corporation, Chouku, Kobe, Japan). C-reactive protein was measured in serum with radial immunodiffusion with fixed-point immune rate methodology (with normal range < 5 mg/L). 

### 2.3. Genotyping

Genomic DNA was extracted using standard phenol-chloroform extraction protocol. Extracted DNA was dissolved in water. We used SNP genotyping assay C_11503414_10 for rs2066865. Genotyping was performed by Taqman Pre-Designed SNP Genotyping Assays (Applied Biosystems, Foster City, California, USA) [18] on a Viia7 Real-Time polymerase chain reaction (PCR) system (Applied Biosystems) according to the supplier’s recommendations.

### 2.4. Data Statistical Analysis

We compared variables with independent-sample (unpaired) *t*-tests by using the SPSS 23 Statistics software (IBM Korea, Seoul, Korea). The Kolmogorov–Smirnov test was used to check whether the variables followed a normal distribution. Normally distributed, continuous variables were presented as mean ± standard deviation (M ± SD) and categorical variables as percentages (%). In case values did not follow a normal distribution, the medians and interquartile ranges (IQRs) were presented. Odds ratios and 95% confidence intervals were calculated to evaluate factor impacts between groups. Comparisons between genotype groups were performed with Kruskal–Wallis H tests for nonparametric variables and with ANOVA for parametric variables. Pearson’s *x*^2^ correlation coefficient and *p* values were calculated, and Spearman’s rank correlation coefficient was used where applicable. Statistical significance was assumed as two-tailed *p* < 0.05.

## 3. Results

### 3.1. Clinical Course 

In total, 104 consecutive patients scheduled for microvascular flap surgery were subjected to analysis after inclusion and exclusion criteria were met. We analyzed 86 males and 18 females classified according to rs2066865 in the *FGG*-carrying genotype: G/G, *n* = 79; G/A, *n* = 22; and A/A, *n* = 3. The genotype results of rs2066865 in the *FGG* A/G polymorphism were all in Hardy–Weinberg equilibrium. The characteristics of the studied group are listed in Table 1 according to the carried genotype of rs2066865 in *FGG*. 

Flap thrombosis was detected in 11.5% of patients (*n* = 12). After unsuccessful salvage, re-anastomosis total flap necrosis eventuated in 7.7% (*n* = 8) of patients, and partial flap necrosis occurred in 4.8% (*n* = 5) of patients, resulting in a microvascular flap survival rate of 92.3% (Table 2).

### 3.2. The relationship between single nucleotide polymorphism rs2066865 in the FGG gene and free flap thrombotic complications.

A higher incidence of flap thrombosis was detected in homozygous A/A and heterozygous A/G genotypes of SNP rs2066865 in *FGG* carriers compared to the G/G genotype carrier (33.3%; 22.7% vs. 7.59%) respectively (OR 0.3 CI, 95% 0.044 to 0.57, *p* = 0.016, Table 3). A risk ratio analysis showed that patients carrying A/A and G/A genotypes were 3.2 times more likely to have flap thrombosis compared to G/G genotype holders.

### 3.3. The Relationship between Single Nucleotide Polymorphism rs2066865 in the FGG gene and Inflammatory Parameters and Platelet Count.

Although we observed a positive association between C-reactive protein and increased plasma fibrinogen concentrations (*r* = 0.580, *p* = 0.002), white blood count, C-reactive protein, and platelet count parameters did not differ between G/G, G/A, and A/A genotype carriers. A/A carriers showed a tendency to have higher plasma C-reactive protein levels without reaching significance (G/G 18.39 vs. G/A 17.62 vs. A/A 31.00 (mg/L); *p* = 0.372).

### 3.4. The Relationship between Single Nucleotide Polymorphism Rs2066865 in the FGG Gene and the Plasma Fibrinogen Level.

**Figure 1:**
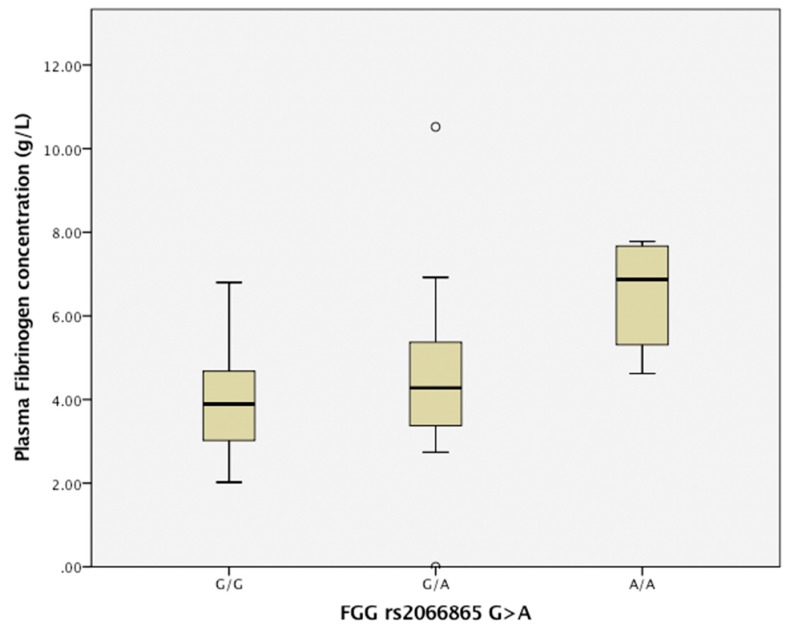
Comparison of plasma fibrinogen concentration in patients with single nucleotide polymorphism rs2066865 in the *FGG* gene (G > A); values are median (IQR range). Genotype A/A carriers had a higher plasma fibrinogen concentration compared to G/A genotype carriers (*p* = 0.003) and compared to G/G genotype carriers (*p* = 0.001).

Patients with SNP rs2066865 in *FGG* gene carriers of A/A and G/A genotypes had higher levels of plasma fibrinogen concentrations (G/G 3.9 (IQR 4.76–3.04); G/A 4.28 (IQR 5.38–3.18); A/A 6.87 (IQR 8.25–5.49); A/A vs. G/A, *p* = 0.003; and A/A vs. G/G, *p* = 0.001), as shown in Figure 1. 

## 4. Discussion

We evaluated the association between a single nucleotide polymorphism rs2066865 in the *FGG* gene, alteration in plasma total fibrinogen concentration, and thrombotic events in microvascular flap surgery.

In our studied group, patients with a variety of injury-causing factors underwent microvascular flap surgery. Overall, our results are within the range of microvascular flap survival rates reported in literature. Shechter et al. analyzed two groups of patients with breast cancer and radiation therapy who underwent postmastectomy breast reconstruction with a deep inferior epigastric artery perforator (DIEP) flap. They reported a total of 8.3% flap loss and vascular anastomosis failure in 5.6% of patients with additional boost radiation compared to zero percent for standard post mastectomy radiation therapy. They concluded that added radiation therapy potentially increased the risk for surgical complication [19]. Bendon et al. retrospectively analyzed patients with lower limb trauma and acute lower limb reconstruction. They had 3 out of 48 (6.25%) patients with flap failure. After revision of arterial and venous anastomosis, the primary complication was due to venous thrombosis in two cases within the first postoperative day [20].

An increased plasma fibrinogen concentration has been proposed to reflect the inflammatory state [11], and inflammation is a well-established risk factor for arterial thrombosis [4]. Particularly, γ’ fibrinogen, one of the fibrinogen molecule chains, has shown a strong association with inflammation. Studies showed that γ’ fibrinogen was highly associated with C-reactive protein levels. In a similar pattern, γ’ fibrinogen levels were elevated during the acute phase and decreased with convalescence time [9]. We found an overall positive correlation between elevated plasma fibrinogen concentration and C-reactive protein, except in homozygous (A/A) SNP carriers, where high fibrinogen levels were significantly associated with increased levels of white blood count, whereas no association with C-reactive protein levels was established.

Interestingly, the reduced levels of fibrinogen γ’/γ and reduction in the γ’/γ ratio were strongly associated with an increase in venous thrombosis. In a study by Uitte de Willige et al., five haplotypes were identified in the *FGG* gene. None of the haplotypes was associated with alterations in the total fibrinogen level, although one of the haplotypes, H_2_, was strongly associated with reduced fibrinogen γ’ levels and a reduced γ’/γ ratio, and both markers were associated with increased risk for venous thrombosis. This indicates that only one *FGG* H_2_ haplotype increases the risk of venous thrombosis by reducing fibrinogen γ’ levels [5]. In our study, because of a lack of resources, we could not perform haplotype reconstructions for the determined polymorphism to compare the data.

Rosendaal et al. found correlation between increased age and changes in fibrinogen levels and an increased risk for thrombosis mainly of venous origin [8]. We found an increase in mean age in patients with homozygosity (A/A) and high plasma fibrinogen levels, though no correlation with an increase in flap thrombosis rate was observed within this group.

The study by Hollenbeck et al. presented data about the role of preoperative platelet count as a predictor of free flap thrombosis. They evaluated a total of 565 acute trauma patients who underwent lower extremity free tissue transfer, and they concluded that acute trauma patients with elevated preoperative platelet counts were at an increased risk for lower extremity free flap thrombotic complications [21]. In contrast, we did not find a significant association between an increase in platelet count and the rate of free flap thrombosis neither in homozygous nor heterozygous SNP carriers.

There were a few limitations in this study. First, as this is a clinical, observational case series study, our sample size was rather small for genetic study. In the Centre of Plastic and Reconstructive Microsurgery of Latvia, around forty microvascular flap surgeries are performed per annum; therefore, we were not able to strengthen our finding by means of genotype intergroup analysis, demonstrating that A/A carriers were associated with an increased risk of microvascular flap thrombosis. In addition, development of thrombosis is multifactorial, including a patient’s related factors and other gene mutations, such as factor V Leiden and prothrombin gene mutation in the heterozygous state, which both present the most common hereditary thrombophilias but possess a relatively low risk for venous thrombosis. Risk increases in the presence of other risk factors (e.g., trauma, major surgery, immobilization, etc.). Deficiencies in antithrombin, protein C, protein S, factor V Leiden, and prothrombin gene in the homozygous state hold greater risk for venous thrombosis, but these mutations are considerably rarer. The observed patients and their family members had a positive history of thrombotic events. As we were focusing on a particular SNP, we were not able to exclude the role of other gene mutations. To add, many patients harbor one or more hereditary or biological risk factors that are not recognized with available methods, and venous thrombosis due to biological causes is found in up to 16.93% of unselected patients [22]. It would be essential to observe gene combinations and possible interactions, which should be part of further investigation.

Secondly, our local perioperative guideline suggests administration of Dextran 40 because of its rheological properties in all patients at the time anastomosis is assured. Thus, its contribution to outcomes in our studied group should be kept in mind. The multiplicity of Dextran 40 effects is governed either by the ability to expand the volume of plasma and, therefore, reduce the hematocrit, which in turn lowers blood viscosity, and, on the other hand, by inhibiting the formation of erythrocyte aggregates as reported by Rosenblum in *Nature* (1968) [23]. In the study reported by Robles et al. [24], Dextran 40 was used to investigate its antithrombotic properties, particularly on platelet function in patients with peripheral artery disease (PAD). They found no difference in spontaneous platelet aggregation and agonist-induced platelet aggregation in response to an increasing Dextran 40 concentration in vitro; however, in patients with known PAD, collagen-induced platelet aggregation and adenosine diphosphate induced aggregation were significantly lower. Whether it plays a role in inhibiting the function of fibrinogen is also not clear. Notably, it has an effect in reducing the density of the fibrinogen network by means of blood volume expansion, thereby modifying blood rheology. In addition, recent data show that the molecular weight of the volume expanders mainly determines the duration of intravascular persistence, and it is not the determining factor in comprising coagulation [25].

Another limitation of the study is the multifactorial nature of the thrombotic event and the difficulty in analyzing the variables separately. Patient-related factors for both arterial and venous thrombosis are described in arterial and venous risk assessment scores, CHAD2S2-VASc and Caprini scores, respectively. In microvascular flap thrombosis, both arterial and venous pools play a role; therefore, we were not able to define an appropriate score for patient thrombotic risk assessment. However, it has been mentioned that the 2005 Caprini Risk Assessment Model could also be applied to patients undergoing microvascular flap surgery, although its ability to predict microvascular thrombosis has not been evaluated [26].

And finally, the patients were localized in the trauma department, and during the postoperative period, a lack of critical clinical monitoring of flap perfusion provoked late readmission to the operating theater, resulting in a 40% salvage rate after revision of anastomosis.

## 5. Conclusions

The results demonstrate that A/A and G/A genotype carriers of a single nucleotide polymorphism in rs2066865 in the *FGG* gene have higher plasma fibrinogen concentrations, and this might be associated with an increased free flap thrombosis incidence rate.

The determined polymorphism could be considered as a genetic marker associated with microvascular flap thrombosis development. Patients that have the determined polymorphism could benefit from perioperative thromboprophylaxis. To confirm the results of this study, the data should be replicated in a greater sample size.

## Figures and Tables

**Table 1:** Group characteristics according to the presence of the determined SNP (*n*, %).

*SNP* rs2066865 (G > A) In the *fibrinogen gamma chain* (*FGG)* Gene	G/G *n* = 79	G/A *n* = 22	A/A *n* = 3
Age (M ± SD)	39.62 (13.03)	46.21 (12.49)	52.0 (14.39)
Sex, female	7 (8.8)	4 (18.1)	1 (33.3)
Free flap thrombosis	6 (7.59)	5 (22.7)	1 (33.3)
History of thrombosis:	2 (2.53)	3 (13.6)	2 (66.7)
arterial (MI; CI)	2 (2.53)	-	
venous (DVT; PATE)	-	3 (13.6)	2 (66.7)
Family history of thrombosis	7 (8.9)	3 (13.6)	2 (66.7)
arterial (MI; CI)	5 (6.37)	-	-
venous (DVT; PATE)	2 (2.53)	3 (13.6)	2 (66.7)
Medication			
antithrombotics	2 (2.53)	1 (4.5)	3 (100)
oral contraceptives	-	1 (4.5)	-
Smoking	22 (27.8)	15 (68.1)	3 (100)
Metabolic disturbances ∗	8 (10.1)	2 (9.1)	1 (33.3)
Alcohol abuse	6 (7.6)	1 (4.54)	1 (33.3)
Defect etiology: trauma	32 (41)	12 (54.5)	43(100)
recent trauma, (<30 d)	22 (27.8)	8 (36.3)	2 (66.7)
polytrauma	6 (7.6)	2 (9.1)	-
chronic inflammation	15 (19)	10 (45.5)	1 (33.3)
malignancy	4 (5.1)	4 (18.2)	1 (33.3)
burn	1 (1.3)	1 (4.5)	-

∗ Diabetes mellitus; adipositas (BMI > 25); MI—myocardial infarction; CI—cerebral infarction; DVT—deep vein thrombosis; and PATE—pulmonary artery thromboembolism.

**Table 2:** Microvascular flap thrombosis needing readmission to the operating theater (*n* = 12).

Flap	Etiology	Defect Localization	Time to Thrombosis	Thrombosis Venue	Re-Anastomosis	Flap Necrosis	Salvage Measure
Scapular/Parascapular	osteomyelitis	lower extremity	18 h	venous	yes	no	additional vein anastomosis
Medial plantar artery flap	osteomyelitis	lower extremity	no data	arterial	yes	no	additional vein anastomosis
LAF	trauma	lower extremity	23 h 55 min	venous	yes	no	additional vein anastomosis
Scapular/Parascapular	osteomyelitis	lower extremity	23 h 30 min	venous	yes	no	-
Scapular/Parascapular	polytrauma	lower extremity	23 h 15 min	venous	yes	yes	additional vein anastomosis
Sartorius free flap	trauma	lower extremity	no data	arterial and venous	no	yes	NPWT; STSG
Osteocutaneus FF	trauma	lower extremity	120 h	arterial and venous	yes	yes	local muscle flap and STSG
Osteocutaneus FF	osteomyelitis	lower extremity	144 h	arterial and venous	yes	yes	local flap
Serratus anterior muscle flap	osteomyelitis	lower extremity	74 h 20 min	arterial and venous	yes	yes	NPWT; STSG
RFF	malignancy	orofacial	13 h 10 min	arterial	yes	yes	local flap
LAF	trauma	upper extremity	no data	venous	yes	yes	ALT flap
Scapular/Parascapular	trauma	lower extremity	49 h	venous	no	yes	local flap; STSG

NPWT—negative pressure wound therapy; STSG—split thickness skin graft; LAF—lateral arm flap; FF—fibula flap; RFF—radial forearm flap; and ALT—anterolateral thigh flap.

**Table 3:** Association of rs2066865 in the *FGG* gene with free flap thrombosis.

SNP (Gene)	Genotypes	Thrombosis Group	Non-Thrombosis Group	OR (CI, 95%) ^a^	*p*
rs2066865 in *FGG*	GG/GA/AA	6/5/1	73/17/2	0.30 (0.044 to 0.57)	0.016

^a^ OR and p value were calculated using logistic regressions adjusted for sex and age, history of smoking, and metabolic disturbances.
